# Predictions From Evolutionary Theory for Urban Environments

**DOI:** 10.1111/eva.70275

**Published:** 2026-06-01

**Authors:** Ailene MacPherson, Aude E. Caizergues, Paul Savary, Kuangyi Xu, Philipp W. Messer, Mia N. Akbar, Marie‐Josée Fortin, Robert D. Holt, Amber Gigi Hoi, Nicole Mideo, Rob Ness, Pedro R. Peres‐Neto, James S. Santangelo, Marc T. J. Johnson

**Affiliations:** ^1^ Department of Mathematics Simon Fraser University Burnaby British Columbia Canada; ^2^ Department of Biological Sciences Simon Fraser University Burnaby British Columbia Canada; ^3^ Department of Biology University of Toronto Mississauga Mississauga Ontario Canada; ^4^ Centre for Urban Environments University of Toronto Mississauga Mississauga Ontario Canada; ^5^ Department of Biology Concordia University Montreal Quebec Canada; ^6^ CNRS, ThéMA Université Marie et Louis Pasteur Besançon France; ^7^ Department of Ecology and Evolutionary Biology University of Toronto Toronto Ontario Canada; ^8^ Department of Computational Biology Cornell University Ithaca New York USA; ^9^ Department of Biology University of Florida Gainesville Florida USA; ^10^ Department of Biology University of Ottawa Ottawa Ontario Canada

**Keywords:** anthropocene, anthropogenic disturbance, urban ecology, urban ecosystem, urban evolution

## Abstract

Urbanization drives rapid and extreme environmental change, profoundly shaping the ecology and evolution of populations. In this Perspective, we call for the integration and development of evolutionary theory and empirical research through collaboration between theoretical and experimental biologists to provide new insights into urban evolutionary ecology. We argue that mathematical models derived from ecological and evolutionary theory can be tailored to provide a powerful framework for generating predictions that can guide empirical research in urban ecology and evolution. At the same time, empirical results can motivate and inform the development and analysis of new theoretical models specific to urban systems. We illustrate how existing evolutionary theory can be harnessed to generate specific predictions of how urbanization can influence evolution. These predictions span the range of urban impacts on all main evolutionary processes, including mutation, gene flow, genetic drift, non‐random mating, and selection. We provide a summary of evidence supporting each prediction and outline empirical approaches available to test them. Importantly, these predictions require distinct modeling approaches that can be applied more broadly to better utilize theory for research on urban environments. To facilitate this, we provide an overview of these existing modeling approaches ranging from the application and syntheses of classic model results to the development of novel probabilistic predictions. We advocate for increased integration of theoretical and empirical research through the development of novel models using parameterization specific to urban systems, empirical tests grounded in theoretical models, model‐based empirical tests, and model‐based data analysis and inference to advance our understanding of evolution in urban environments.

## 1 The Importance of Urbanization for Evolution

Urbanization drives rapid environmental change, profoundly impacting populations of many taxa, threatening native biodiversity, and homogenizing ecological communities (Grimm et al. 2008; Hahs et al. 2009; Filazzola et al. 2024). Urban areas constitute unique, human‐modified ecosystems where dramatic shifts in environmental conditions and landscapes directly influence evolution by increasing mutation rates (Johnson et al. 2024), amplifying genetic drift and altering gene flow (Miles et al. 2019), changing mating systems (Rivkin and Johnson 2022), and shifting the strength and direction of selection (Charmantier et al. 2024). While urbanization affects the same evolutionary processes that can be studied in any environment (Johnson and Munshi‐South 2017), evidence is emerging that urban development and the environments particular to cities, present unique evolutionary challenges to organisms (Szulkin et al. 2020). The pace and magnitude of urban environmental change are hypothesized to alter the relative importance of different evolutionary mechanisms compared to more natural environments (e.g., increasing the effects of genetic drift). These shifts in evolutionary process can, in turn, lead to novel outcomes that can in turn shape the ecology of populations (e.g., the persistence of some populations and the extirpation of others (Alberti 2024)). In this Perspective, we argue that understanding the effects of urbanization on evolutionary processes and patterns will benefit from interdisciplinary collaboration aimed at integrating ecological and evolutionary theory and empirical work to provide new insights into urban evolutionary ecology.

As urban evolutionary biology has developed over the past decade, conceptual theories have been proposed to make general predictions about evolutionary responses to urbanization (Alberti 2015; Johnson and Munshi‐South 2017; Miles et al. 2019; Szulkin et al. 2020; Diamond and Martin 2021; Johnson et al. 2024). Yet, the vast body of mathematical theory developed in evolutionary biology has seen little direct application that would allow for specific empirical predictions in urban environments. Cities can serve as an empiricist's “large‐scale ‘experimental’ mesocosm” for exploration of evolution in response to rapid anthropogenic environmental change. To an empiricist, the scale, global replication, and magnitude of environmental changes offer an amazing opportunity to test theoretical models in a context that can provide both fundamental and applied insights. In a similar way, cities can provide a valuable and arguably unique testing ground for theoretical evolutionary biologists. The rapid, large and transient dynamics of urban environmental change may require theoreticians to reimagine the temporal scale and parameter space of models and shift their focus to non‐equilibrium dynamics that may vary across cities and over time. The value of theoretical models tailored to urban systems would be multi‐fold; they would provide proof‐of‐concept validation of conceptual theories and help identify hidden assumptions of these theories and counterintuitive evolutionary responses to urbanization (Servedio et al. 2014); they could connect urban patterns to specific evolutionary processes (Shaw et al. 2024); and they would identify general principles that extend across urban systems. When tailored specifically to cities (Santangelo et al. 2018; Savary et al. 2024; see Box 1), mathematical models can guide empirical work by helping to formulate and assess hypotheses and predictions. We contend that the development of models and theory specific to the spatial and temporal scale of urban systems could yield conceptual and applied benefits for both theoretical and empirical research.

**FIGURE 1:**
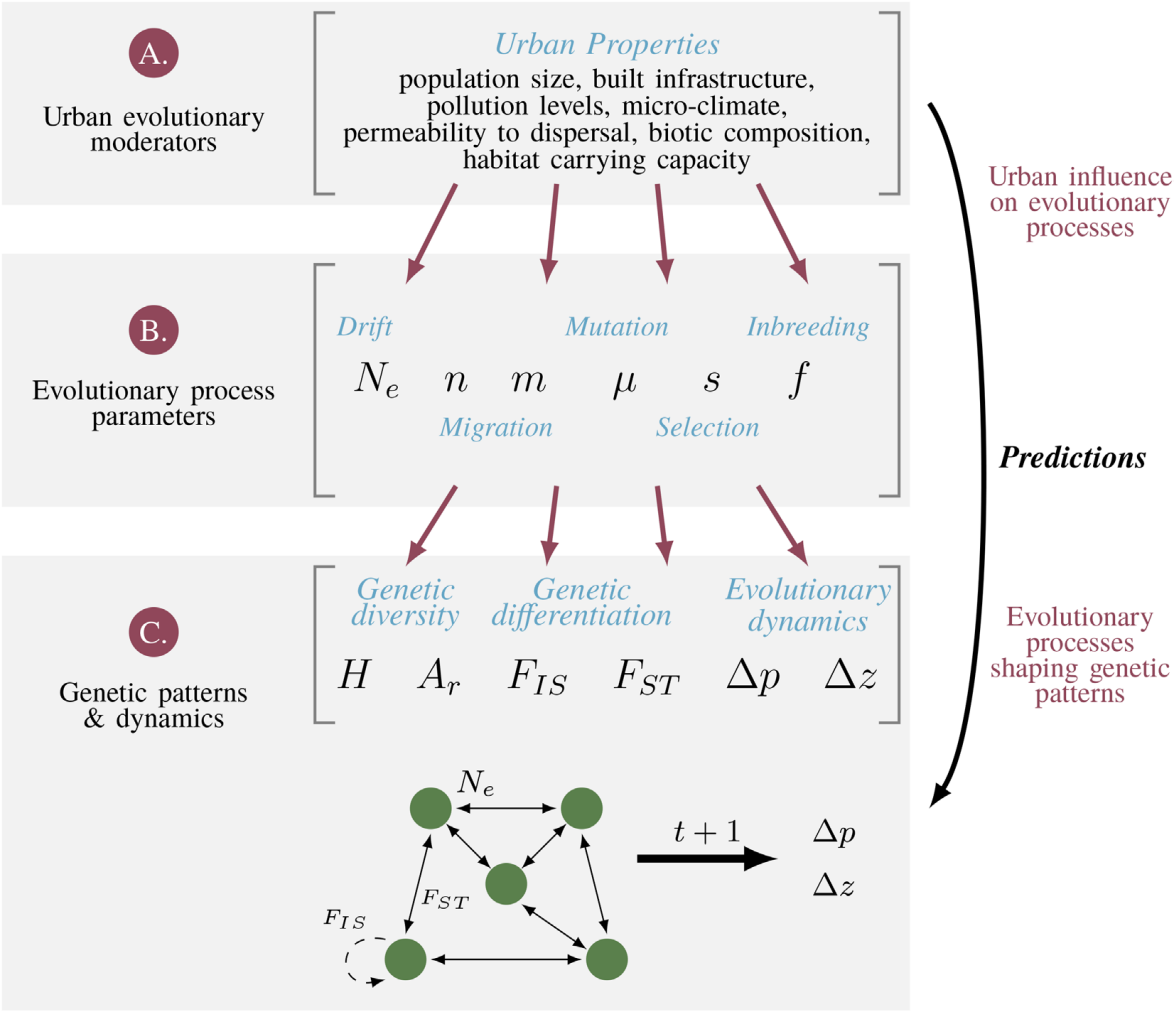
Formulation of theoretical predictions for urban evolution. The properties of urban environments (Panel A) alter evolutionary processes (Panel B) via changes in effective population size, $N_{e}$, habitat fragmentation as determined by the number of habitat patches n and gene flow m, mutation rate μ, inbreeding f, and the strength and direction of selection s. Changes in these evolutionary parameters determine the observed genetic patterns in urban environments (Panel C), shaping genetic diversity (as measured by expected heterozygosity H or allelic richness $A_{r}$), genetic variation within and between interconnected habitat patches ($F_{\text{IS}}$ and $F_{\mathrm{ST}}$), and evolutionary dynamics (changes in allele frequency ∆p and phenotype ∆z). Green circles in Panel C indicate habitat patches, solid arrows gene flow, and the dashed arrow the effect of non‐random mating (e.g., inbreeding).

This article emerged from a workshop that brought together evolutionary biologists working in urban systems and theoreticians with a broad range of expertise across ecology and evolution. Our Perspective reflects our shared view on the benefits of interdisciplinary collaboration between theorists and empiricists working through multiple points of connection, contention, and confusion during and after the workshop. In writing this article, we aim to provide guidance on how to bridge the gap between theoretical and empirical research, addressing two key audiences. For urban empiricists, we provide a primer on a curated sample of evolutionary theories that can be tailored to urban systems to offer testable predictions for future studies of urban evolutionary ecology. For evolutionary theorists, we pinpoint urgent questions in the emerging field of urban evolution to elicit the development of theoretical models tailored to these globally replicated, human‐modified ecosystems. We demonstrate how theoretical models can contribute to urban evolutionary biology by presenting five predictions drawn from the existing theoretical literature that address a broad array of evolutionary questions. These predictions examine how urban environments influence the main evolutionary processes (Figure 1, Table 1)—mutation, drift, gene flow, non‐random mating, and selection. Through these predictions, we aim to clarify the complex links between urban characteristics, evolutionary processes, and the genetic and ecological patterns they ultimately shape. While these predictions serve only as a starting point, we hope that the intentional integration of empirical and theoretical perspectives offers a constructive framework and foundation for future advances in urban evolutionary biology.

**TABLE 1:** Summary of evolutionary processes and predictions.

Evolutionary process	Effect of urbanization on evolutionary processes	Prediction	Theoretical approach
**Mutation** and genetic drift	Urbanization is expected to increase mutation rates.	*Elevated mutation rates in urban environments will gradually increase genetic diversity over thousands of generations. This increased genetic diversity can be a source of adaptive variation and increased genetic load, particularly for species with short generation times*.	Complementing the analyses of the equilibria of **dynamical models**, following transient dynamics after an urbanization‐driven perturbation from equilibrium can inform the timescale and potential importance of evolutionary processes in young urban landscapes.
**Genetic drift** and gene flow	Urbanization is generally expected to fragment landscapes, alter the permeability and spatial distribution of habitat patches, and reduce census population sizes.	*Changes in population size, habitat fragmentation and loss, and environmental permeability will determine whether urbanization leads to a significant erosion of genetic diversity, putting inbred populations at risk of local extirpation*.	**Simulation** methods can characterize evolutionary patterns in urban systems when multiple interacting evolutionary processes (e.g., migration, selection, and drift) are involved or, as in Savary et al. (2024), when the structure of the urban landscape is complex.
**Non‐random mating** and selection	Urban populations are often the result of recent colonization events. Urban landscapes can reduce reproductive assurance (e.g., through the loss of pollinators).	*Selection for selfing, and other forms of inbreeding, will be stronger in urban populations than in non‐urban ones, particularly when inbreeding depression is driven by recessive alleles*.	In a similar manner to urban–rural contrasts, explicit **model contrasts** (e.g., between models with vs. without population subdivision) can elucidate the effect of key ecological and evolutionary features.
**Selection** and mutation	Urbanization can result in abrupt changes in the fitness landscapes shifting phenotypic optima.	*When adaptation to urbanization involves de novo mutations, these mutations will be of relatively large phenotypic effect, especially during the early stages of the adaptive walk towards the new fitness optimum*.	**Fitness landscapes**, such as Fisher's Geometric Model, can be applied and parameterized to formulate predictions for urban adaptation.
Selection and drift	Key urban environmental conditions are often replicated across cities allowing a naturally replicated structure.	*Parallel adaptive genetic evolution at a locus is most likely to occur if the focal allele is strongly beneficial relative to drift and/or if the focal allele is already at high frequency before urbanization*.	The naturally replicated nature of urban environments makes them ideal for testing predictions from **probabilistic models**. For example, results derived from diffusion approximations (e.g., allele frequency distributions, fixation times or probabilities).

The five predictions also showcase distinct theoretical approaches for studying urbanization. These approaches range from the application of classic modeling frameworks to the development of system‐specific simulation‐based approaches. Paralleling these theoretical approaches, we highlight the range of empirical approaches (e.g., urban–rural contrasts, parallel evolution across cities, comparative evolution) necessary to test these predictions. We conclude with a call to theoreticians, highlighting particular aspects of urban evolution that would benefit from future theoretical exploration.

## 2 Theoretical Predictions

### 2.1 Effects of Urbanization on Mutation

Urban environments are often mutagenic. Industrial pollution (an example of an urban evolutionary moderator, see Figure 1A) has been linked to a two‐fold or more increase in mutation rates (Johnson et al. 2024) in birds (Yauk et al. 2000; Somers et al. 2002), mammals (Somers et al. 2004; Arif et al. 2024), and bacteria (White and Claxton 2004). Given that mutation is the ultimate source of genetic variation, a comprehensive understanding of evolution in urban environments requires a description of how genetic diversity changes in response to increased mutation. Here we ask, does an increase in mutation rate (an evolutionary process parameter, see Figure 1B) provide a source of variation on a time scale relevant to urban adaptation? One measure of genetic diversity in a population is expected heterozygosity, H–the probability that two alleles at a locus drawn at random from a population are different (a genetic pattern, see Figure 1C). At Hardy–Weinberg equilibrium in a diploid population, expected heterozygosity is equal to the observed frequency of heterozygotes.

**FIGURE 2:**
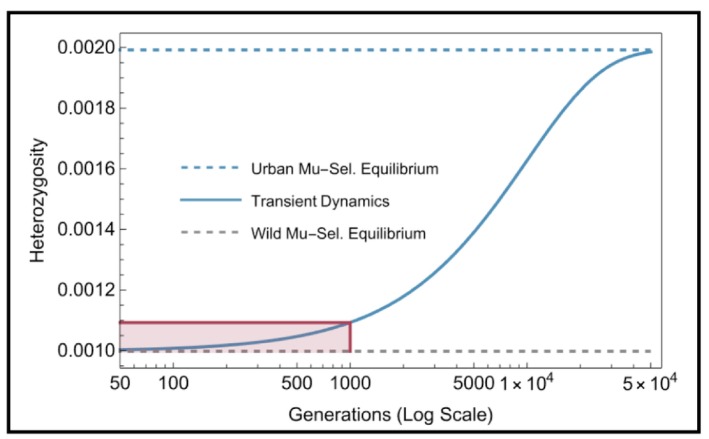
Effect of mutation on heterozygosity. Dynamics of expected heterozygosity (solid blue curve) following an increase in mutation rate from the wild equilibrium (gray dashed) to the urban equilibrium (blue dashed). Red lines and shaded area indicate the 9.5% increase in heterozygosity after 1000 generations. Generations shown on a log‐scale to emphasize both the transient behaviour expected in many urban systems as well as long‐term dynamics. Parameters: $\mu_{\text{wild}}=10^{-7},\mu_{\text{urban}}=2\mu_{\text{wild}},\text{and}\;N_{e}=10^{4}$.

One way to model mutation‐drift balance is with the Wright‐Fisher model—a model with non‐overlapping generations and a constant (effective) population size $N_{e}$. In this model, the expected heterozygosity in the wild population is given by $H^{\prime }=\frac{N\mu_{\text{wild}}}{1+2\left(N_{e}-1\right)\mu_{\text{wild}}}$, where $\mu_{\text{wild}}$ is the historical mutation rate in the non‐urban environment. This equilibrium genetic diversity can be obtained from a recursion equation for mutation‐drift balance (see modeling Approach 1 below). If urbanization results in an increase in the mutation rate (e.g., $\mu_{\text{urban}}>\mu_{\text{wild}}$), this same recursion equation can be used to follow the resulting increase in heterozygosity (Hartl and Clark 2006, Equation 6.8):

(1) $$H\left(t+1\right)=\mu_{\text{urban}}+H\left(t\right)\frac{\left(N_{e}-1\right)\left(1-2\mu_{\text{urban}}\right)}{N_{e}}$$

until the population reaches the new equilibrium. The resulting transient dynamics of expected heterozygosity—the gradual increase in heterozygosity as the population shifts from its historical wild equilibrium to the new urban one—is shown in Figure 2, and leads to our first prediction:

Prediction 1 *Elevated mutation rates in urban environments will gradually increase genetic diversity over thousands of generations. This increased genetic diversity can be a source of adaptive variation and increased genetic load, particularly for species with short generation times*.

The change in heterozygosity over time is both slow and non‐linear. For example, as shown in Figure 2, a doubling of the mutation rate from its historical level ($\mu_{\text{urban}}=2\mu_{\text{wild}}$) results in a ~10% increase in heterozygosity within ca. 1000 generations. In contrast, the urban equilibrium is approached much more slowly (ca. 50,000 generations). Given the long timescales of these dynamics, the effect of mutation rate on overall genetic diversity may mostly affect adaptation in species with short generation times, such as viruses, bacteria, some fungi or water fleas, or for traits that are polygenic.

Many mutations are deleterious, and one might anticipate that an increase in mutation rate would increase genetic load, potentially hampering the persistence of small populations in a wide range of organisms, even with small increases in heterozygosity. An important question moving forward is: How do increased mutation rates in cities affect evolutionary potential and evolutionary rescue versus genetic load and extinction risk in urban environments? We see the potential for a fruitful collaboration between theoretical and empirical researchers in answering this question—one that involves modeling and experimentally testing the rate and magnitude of the effects of urban pollution on the accumulation of deleterious versus adaptive mutations throughout the genome, as well as their ecological consequences.

### 2.2 Effects of Urban Habitat Distribution on Genetic Drift and Gene Flow

The extent of standing genetic diversity in populations, and hence the evolutionary potential of a population, is determined by the balance between the generation of diversity through mutation (Prediction 1) and its loss through genetic drift. In addition to altering mutation rates, urbanization can impact drift via broadscale or temporal changes in (census) population size, $N_{c}$, habitat fragmentation, environmental permeability, and the spatial configuration of habitat patches (Miles et al. 2019; Munshi‐South and Richardson 2020) (Figure 1A). The cumulative effect of urbanization on genetic drift is captured by the effective population size, $N_{e}$, the size of a theoretically‐ideal population that loses variation through genetic drift at the same rate as the focal population (Figure 1B). Specifically, theory predicts a loss of genetic diversity at a rate proportional to $\frac{1}{N_{e}}$, such that decreasing $N_{e}$ increases the strength of genetic drift (Figure 1C). By examining how the properties of urban environments impact $N_{e}$, simple theoretical models can help answer the question: Under what conditions do we expect urbanization to increase or decrease genetic diversity, and how does this answer depend on the spatial scale at which genetic diversity is assessed?

**FIGURE 3:**
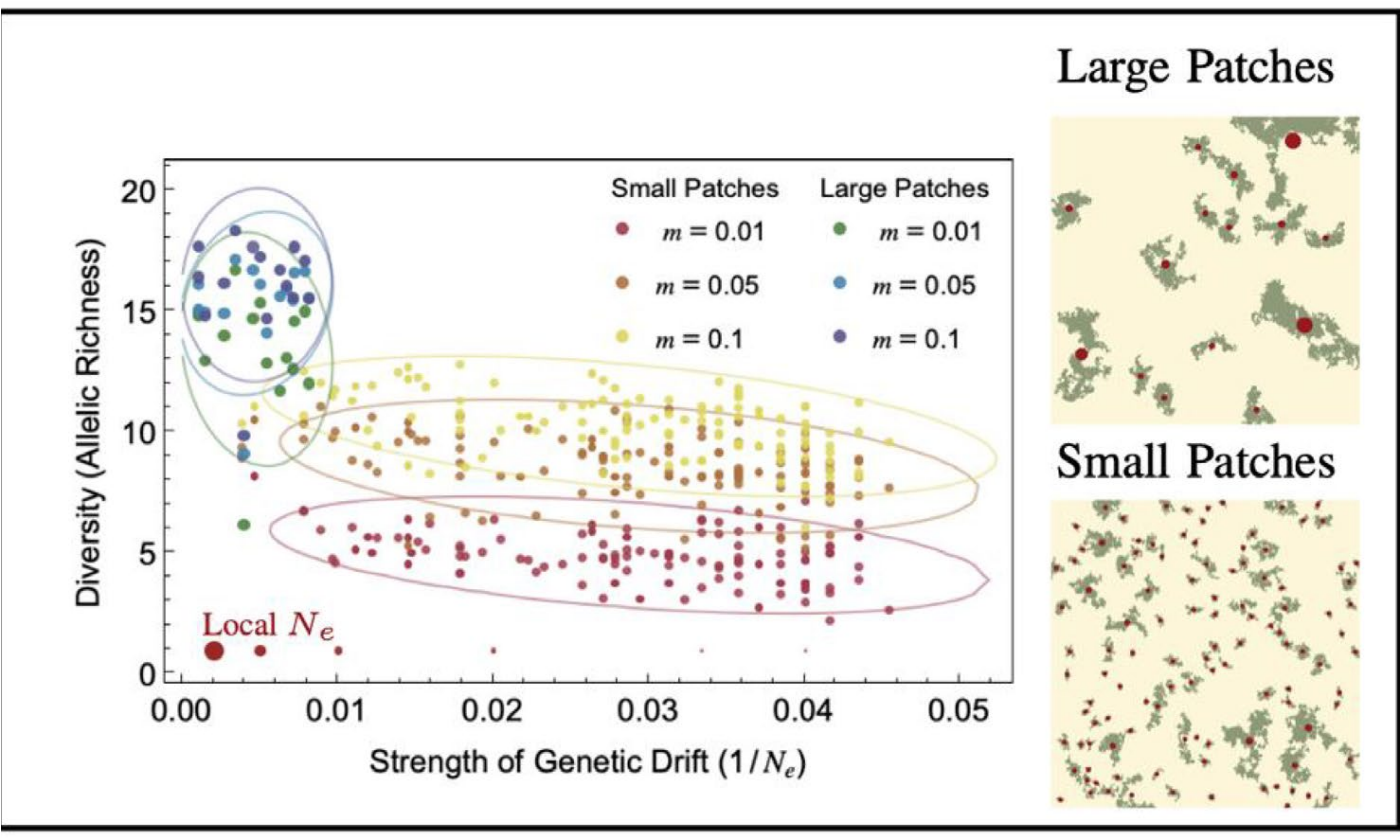
Effect of habitat distribution, drift, and gene flow on allelic diversity. Allelic richness is measured within patches of two urban landscapes fragmented into either large or small habitat patches but with the same total area (right hand panels). Patches are connected through gene flow as per the migration rate m and mediated by the distance between them. The local effective population size $N_{e}$ within habitat patches is assumed to be proportional to local habitat area [Simulations adapted from Savary et al. (2024), for further modeling details see Data Availability Statement].

To understand genetic diversity in cities, we must consider drift both within “local” habitat patches and at the city‐wide “global” scale because theory predicts that diversity will show contrasting patterns across these scales (Hartl and Clark 2006, 126). This can be done with simulations (see modelling Approach 2) in which the population size of each patch, and by extension ‘local’ genetic drift, depends on patch area, and gene flow intensity depends on the migration rate and the distance between patches (Figure 3; Savary et al. 2024; Box 1). First, increased fragmentation, for a given total habitat area, will reduce the size of individual patches and increase the importance of local genetic drift (Figure 3: top vs. bottom landscape). By adapting the simulations in Savary et al. (2024) to follow genetic diversity in fragmented habitats with varying patch size (see Figure 3), we show that the resulting loss of diversity (e.g., as measured by allelic richness) in small urban patches can be significant, with direct consequences on inbreeding depression (see Prediction 3 and Approach 3). Yet, the negative effects of drift on genetic diversity can be counteracted by gene flow (Figure 1c), even if only a small number of immigrants breed within the focal population (Mills and Allendorf 1996; Savary et al. 2024). Modelling results illustrated in Figure 3 show that high migration rates (*m*) among small patches can maintain similar genetic diversity levels as low migration rates among a few large patches. Indeed, there exists considerable empirical evidence of decreased diversity in small isolated urban habitat patches, such as the dramatic loss of diversity in the northern dusky salamander ( *Desmognathus fuscus* ) in New York City (Munshi‐South et al. 2013). By contrast, species that maintain high population sizes and/or high dispersal, do not show this pattern (e.g., white clover ( *Trifolium repens* ) (Caizergues et al. 2024) and feral pigeons ( *Columba livia* ) (Carlen and Munshi‐South 2021)). These effects of drift and gene flow on local diversity lead to the following prediction:

Prediction 2 *Species with low dispersal that experience negative demographic effects of urban environmental change will exhibit a local erosion of genetic diversity that potentially elevates inbreeding, putting inbred populations at risk of local extirpation*.

The substantial variation in population size, habitat connectivity, and green/impervious patch layouts across species and cities provide the basis to model the potential outcomes of genetic drift and gene flow in urban settings and test the resulting predictions with comparative approaches.

Although the effects of urban fragmentation and connectivity on local population diversity have been well studied (Miles et al. 2019; Schmidt et al. 2020, 2022), the effects of these processes on city‐wide genetic diversity have largely been neglected. While fragmentation is expected to decrease genetic diversity locally (in the absence of gene flow), diversity can be maintained city‐wide, at least in the short term, as fragmentation paradoxically leads to a higher global $N_{e}$ (Hartl and Clark 2006, 126). This counter‐intuitive result arises due to the semi‐independence of genetic drift across habitat patches—while diversity declines within each patch, each will retain a distinct sample of the total diversity, thus increasing the diversity among patches (i.e., due to the genetic differentiation among the data points of Figure 3). In the long term however, increased local drift, inbreeding, and local extirpation can lead to the loss of genetic diversity even at this global scale (Frankham et al. 2017). The net effect of genetic drift and gene flow on city‐wide genetic diversity will depend on the network structure and connectivity of habitat patches. Gene flow and drift at the local and global scale can also impact a species' adaptive potential. If fragmentation maintains city‐wide diversity, it can help preserve a pool of genetic variation with which species can adapt to stressful urban environments (Lenormand 2002). Two important questions moving forward are: What types of cities (e.g., size, climate) and urban designs maximize the maintenance of genetic diversity? And does such a design maximize the probability of evolutionary rescue and population persistence?

### 2.3 Effects of Urban Environments on Non‐Random Mating

Plants and animals exhibit a great diversity of reproductive (e.g., sexual, asexual) and mating systems (e.g., outcrossing, inbreeding; Jarne and Charlesworth 1993). Mating systems (Ashman et al. 2014) and sexually‐selected traits (Sepp et al. 2020) can evolve rapidly in response to ecological change. For example, plants in urban environments are often characterized by recent colonization events, high fragmentation, harsh conditions, and reduced pollinator diversity (Wenzel et al. 2020; Baldock 2020), all of which can promote the evolution of self‐fertilization. These ecological changes, however, must be severe enough to overcome selection against selfing due to inbreeding depression, the extent of which will depend on factors such as the pre‐urbanization population size and the architecture of genetic load (Lohr and Haag 2015). This leads to the question: Under what conditions are urban environments most likely to favour shifts in mating systems, such as shifts from outcrossing to inbreeding or self‐fertilization?

Selfing, and other forms of inbreeding, may be directly favoured in urban systems because they increase reproductive assurance when pollinators or mates are limited (Strobeck 1975; Bodbyl Roels and Kelly 2011; Busch and Delph 2012), and when resources available for reproduction are scarce (Sicard and Lenhard 2011; Lehtonen et al. 2012). Urban environments may also act as a filter favouring self‐compatible species. Indeed, the proportion of self‐compatible species is higher in urban compared with rural areas (Desaegher et al. 2019). Theory also predicts that urbanization‐mediated demographic changes, such as frequent colonization events (Kirkpatrick and Jarne 2000) and increased population subdivision (Whitlock 2002), may contribute to mating system evolution within a species. Namely, these changes may reduce inbreeding depression (Charlesworth and Willis 2009), thereby relaxing selection against selfing.

Habitat fragmentation and the resulting population subdivision in urban environments can also decrease inbreeding depression as deleterious and partially‐recessive mutations are either lost by drift or are purged more efficiently if they drift to high frequency within individual sub‐patches. The relative extent of inbreeding depression in a population with and without subdivision (see Approach 3 below) depends both on the extent of genetic differentiation between habitat patches, $F_{\mathrm{ST}}$, (Whitlock 2002) and the dominance, h, of the deleterious mutations as given by the equation:

(2) $$\begin{array}{c} \text{Relative Inbreeding}\\ \text{Depression} \end{array}=\frac{\left(1+F_{\mathrm{ST}}\right)h}{F_{\mathrm{ST}}+\left(1-F_{\mathrm{ST}}\right)h}$$

From these considerations of how urbanization may impact the cost of inbreeding and alter mate availability we can formulate the following prediction:

Prediction 3 *When urban environments decrease the opportunity for mating among individuals, selection for selfing and other forms of inbreeding will be stronger in urban than in non‐urban populations, particularly when inbreeding depression is driven by recessive alleles*.

**FIGURE 4:**
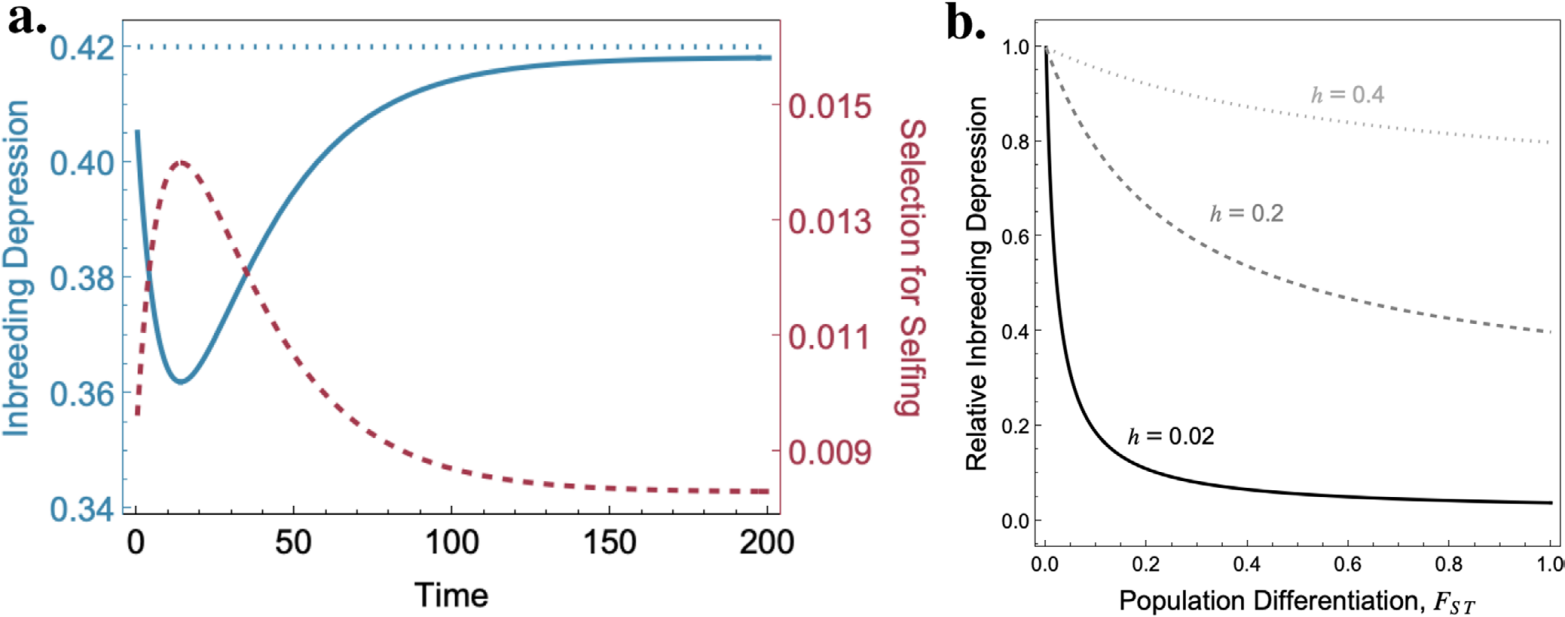
Inbreeding depression and the evolution of selfing following population bottlenecks from urbanization. Panel a: Inbreeding depression as a result of highly recessive lethal alleles (blue curve, h=0.002, s=0.8) following a population bottleneck (of N=10 diploid individuals) and corresponding selection coefficient (red curve) for a modifier allele which increases the selfing rate by 10%. The pre‐bottleneck inbreeding depression is shown by blue dashed line. Panel b: Inbreeding depression in a meta‐population relative to a panmictic population as a function of population differentiation between habitat patches and the degree of dominance h. [Results adapted from (Lande and Schemske 1985; Kirkpatrick and Jarne 2000; Whitlock 2002)].

Consider the case when a wild population initially colonizes or recolonizes an urban habitat. These events create bottlenecks, reducing the population to a few founders. Inbreeding depression is expected to initially drop following a bottleneck because rare deleterious alleles are lost, although it will eventually return to pre‐bottleneck levels (Figure 4A) (Kirkpatrick and Jarne 2000), and its magnitude largely depends on genetic differentiation ($F_{\mathrm{ST}}$) and the dominance of deleterious alleles involved (h) (Figure 4B). Alleles that enhance selfing but that are selected against in wild populations will be temporarily favoured following a bottleneck (Figure 4A) (Lande and Schemske 1985; Cheptou and Donohue 2011). Indeed, in species capable of both outcrossing and self‐fertilizing, urban populations that have undergone bottlenecks should exhibit a higher frequency of selfing than their non‐urban counterparts. Although the few empirical studies available have found little direct evidence of increased selfing rates, they have demonstrated a loss of genetic diversity in urban populations and increased rates of inbreeding (Cheptou and Avendaño 2006).

While bottlenecks and population structure only transiently favour selfing, selfing itself increases the efficiency with which selection purges deleterious alleles, such that, if a sufficiently high selfing rate evolves during initial colonization, inbreeding depression will remain suppressed relative to the ancestral population (Roze 2015). As a result, inbreeding depression may remain suppressed in the long‐term in highly fragmented urban landscapes. A more comprehensive understanding of mating system evolution in urban environments will require additional empirical work to address key questions. For example, how do reproductive systems (e.g., sexual vs. asexual reproduction), mating systems (e.g., outcrossing vs. selfing), and genetic systems (e.g., ploidy, recombination, sex determination) differ between urban and non‐urban habitats, and how does such variation match with theoretical predictions?

### 2.4 Effects of Urbanization on Natural Selection Within and Among Cities

Urbanization is associated with rapid and substantial biotic and abiotic changes including physical, chemical, and biological transformations. The nature and extent of environmental change, and how each population responds to it, varies across cities and species (Charmantier et al. 2024). This complexity of the adaptive landscape can be captured theoretically (MacPherson et al. 2015) in terms of trait dimensionality**—**the number of independent phenotypic traits that experience selection in a particular environment**—**which depends on both the distribution of phenotypes within the population experiencing selection and the environment to which the focal population is adapting. Whether adaptation predominantly involves evolution of a few versus many traits has numerous evolutionary consequences (White and Butlin 2021). In general, urbanization tends to create environmental change that results in profound mismatch between an organism's phenotype and the novel urban environment, whereas most other environmental change scenarios typically occur more gradually and result in weaker mismatch (Cheptou et al. 2008; Rodewald et al. 2011; Halfwerk et al. 2019). Given this context, we first ask: When adaptation to urbanization occurs via new mutations, is adaptation likely driven by many mutations of small effect or a few mutations of large effect? How does this depend on the complexity of the environmental change?

**FIGURE 5:**
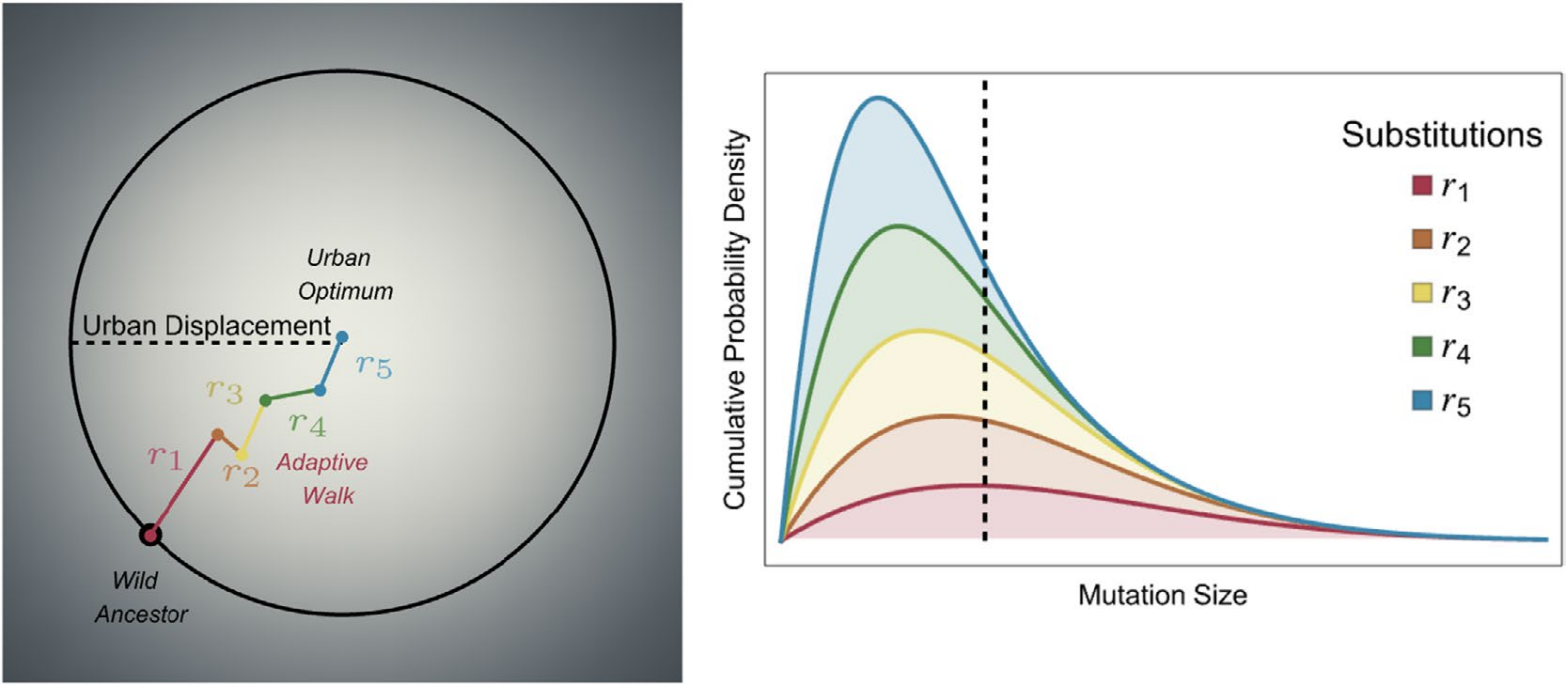
Adaptive walk following urbanization. Left‐hand panel: Adaptive walk (coloured vectors, $r_{1},r_{2},\ldots ,r_{5}$) from a wild ancestor (black point) to the urban optimum. Gray shading indicates the mean population fitness in the urban environment, with lighter colors corresponding to higher fitness values. Right‐hand panel: The net probability density of mutation sizes for the first five substitutions is shown in the inset. Dashed vertical line indicates displacement of the wild population from the new urban optimum. [Results adapted from (Orr 1998)].

Fisher's geometric model is a classic modeling framework (see Approach 4 below) that examines how a population evolves in response to mutations given a geometric depiction of a fitness landscape of two or more traits. It has provided the foundation for many theoretical studies of adaptation and is ideally suited for answering the question outlined above (Fisher 1930). In this model, mutations move phenotypes in random directions (pleiotropy) in multidimensional phenotype space (shown for the 2‐dimensional case in Figure 5), in which the fitness landscape is peaked around a single optimal point in this space. Studies of Fisher's model and its extensions have led to fundamental insights into how populations are expected to adapt after displacement from a fitness optimum (Orr 1998, 2005; Tenaillon 2014; Connallon and Hodgins 2021) following an adaptive walk—the sequential fixation of primarily adaptive mutations**—**as the population transitions from the initial non‐urban state towards the new urban fitness peak. Specifically, Fisher's model leads to the following prediction:

Prediction 4 *When adaptation to urbanization depends on novel mutations, these mutations will be of relatively large phenotypic effect, especially during the early stages of the adaptive walk towards a new fitness optimum*.

A natural question that arises is in what biological systems and cities such a prediction may apply? A famous example of strong selection favouring large effect variants is the evolution of melanism in the peppered moth ( *Biston betularia* ), where a transposable element responsible for melanism conferred an adaptive advantage (i.e., camouflage) following darkening of trees by black soot during the onset of industrialization. The effect of trait dimensionality, in contrast, is more nuanced (MacPherson et al. 2015; White and Butlin 2021). On one hand, populations faced with adapting along multiple environmental axes experience more selection overall, amplifying the above prediction (i.e., fewer mutations but larger effect). On the other hand, as the complexity of the environmental change increases, the more likely it is that a large effect pleiotropic mutation will be mis‐aligned with the environmental shift from urbanization, forcing populations to adapt in a more polygenic manner. Understanding evolution in urban environments therefore requires first clarifying the complexity of environmental change in each case, and empirical studies identifying environmental factors that drive adaptation provide an important foundation for this work. Further empirical studies can then test these predictions by quantifying the net selection on urban populations and characterizing the genetic architecture of adaptation including the number and effect sizes of substitutions underlying adaptation to urban environments and the extent of pleiotropy among them.

While we have focused on de novo mutations in this prediction, an important question to resolve is: What are the relative roles of new mutations versus standing genetic variation in driving adaptation to urban environments? In line with this, for the next prediction, we broaden our discussion of natural selection from adaptation via de novo mutations in urban–rural contrasts to comparisons of the genetics of adaptation among cities from standing genetic variation.

Urbanization often results in similar environmental changes across cities (Santangelo et al. 2022). For example, cities consistently have more impervious surfaces, greater pollution, warmer temperatures, less vegetation, more artificial light at night, and higher human densities (Grimm et al. 2008; Niemelä 2011). Given comparable environments among geographically disparate cities, cities are therefore ideal systems to investigate the conditions that promote or prevent repeated adaptation through parallel evolution**–**the independent evolution of similar genotypes or phenotypes in two or more populations, typically in response to similar environmental changes. A natural question to ask is: How common is parallel evolution due to natural selection, and how does the probability of repeated adaptation depend on the number of cities studied or the relative strength of evolutionary forces (e.g., selection vs. genetic drift) acting in urban environments?

The replicated nature of cities can be leveraged theoretically through the use of probabilistic models (see Approach 5). For example, here we apply the model developed in MacPherson and Nuismer (2017) to formulate predictions for parallel genetic evolution in urban environments by considering the probability that a beneficial allele (with a selection coefficient s) fixes repeatedly and independently in c cities—a probability denoted as $P_{\mathrm{fix}}^{c}$.

(3) $$P_{\mathrm{fix}}^{c}={\left(\frac{1-e^{-N_{e}sp_{0}}}{1-e^{-N_{e}s}}\right)}^{c}$$

As above, $N_{e}$ is the effective population size and $p_{0}$ is the initial frequency of the beneficial allele. This model assumes that all evolutionary parameters ($N_{e}$, s, and $p_{0}$) are the same across all the cities considered. From this probability expression we can formulate the following prediction.

**FIGURE 6:**
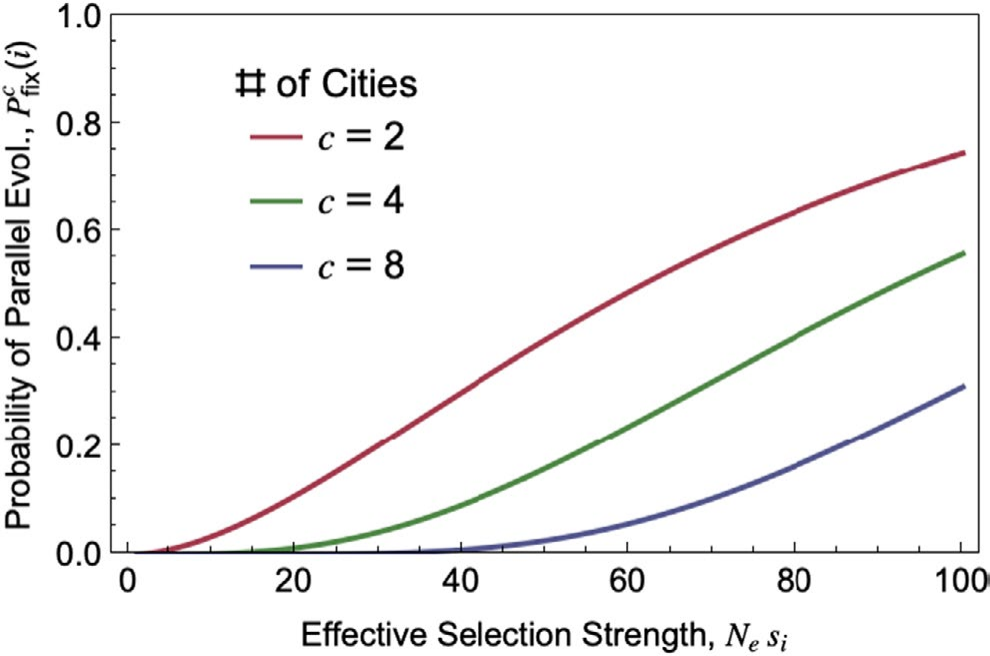
The probability of parallel evolution across cities. The probability a beneficial allele at locus i fixes independently in $c=\left\{2,4,8\right\}$ cities, depends on the effective strength of selection $N_{e}s_{i}$ favouring that allele and the initial frequency of the beneficial allele $p_{0}$ [Results adapted from MacPherson and Nuismer (2017), shown here for the case where $p_{0}=0.01$].

Prediction 5 *Parallel adaptive genetic evolution at a locus is most likely to occur among cities if the focal allele is strongly beneficial relative to drift (N*_*e*_ *s is large) and/or if the focal allele is already at high frequency before urbanization* $\left(0\ll p_{0}\right)$ (Figure 6).

Further elaborations of the probabilistic model in (MacPherson and Nuismer 2017) show that parallel phenotypic evolution is most likely when a phenotype is controlled by fewer loci of large effect (MacPherson and Nuismer 2017; Thompson et al. 2019), consistent with Prediction 4. In line with Prediction 5, hydrogen cyanide production in white clover, which is controlled by two large‐effect loci that are under strong selection in response to urban environments (Santangelo et al. 2022; Martin and Johnson 2025), evolved in parallel from standing genetic variation along multiple independent urban‐to‐rural gradients (Santangelo et al. 2022).

These theoretical predictions bring into focus unanswered questions important to understanding the likelihood of parallel evolution to cities. These questions are: How do urban environments influence the strength and consistency of selection across cities (Charmantier et al. 2024)? What is the genetic architecture of traits that confer adaptations to urban environments in terms of the number of loci, dominance, and epistasis? And how does the relatively young age of cities influence the probability and genetic architecture of parallel evolution within species, or convergent evolution between species?

Future theoretical developments are also needed to examine the extent of parallelism in light of the unique features of urban environments. For example, how do model predictions change when environmental change is fast and multidimensional? How does parallelism depend on the spatial scale at which selection acts and how sensitive is it to changes in the strength of selection over space and time due to ongoing urbanization? Probabilistic models (see Approach 5 below) can be applied at the phenotypic and genetic levels, allowing us to examine the probability of parallel evolution of phenotypes driven by distinct genetic variants. For example, repeated evolution in killifish ( *Fundulus heteroclitus* ) populations for tolerance to toxic aquatic pollution has been linked to strong selection on multiple distinct variants affecting the aryl hydrocarbon receptor‐based signaling pathway (Reid et al. 2016).

### 2.5 Modeling Approaches for Urban Environments

Each of the five predictions exemplifies a distinct modeling approach that can be applied more broadly to formulate additional predictions for evolution in urban environments. Representing different mathematical or conceptual strategies for representing evolutionary processes, these modeling approaches are not mutually exclusive; a comprehensive understanding of evolution in urban environments will likely often require integrating the methods and insights from multiple approaches. These approaches lead to different kinds of outputs (e.g., equilibria, simulated data sets, probability distributions) and hence distinct insights into urban adaptation. We believe that these approaches can be used to further bridge theoretical and empirical thinking in urban evolutionary biology and present them here along with a description of the role each may play in understanding evolution in urban environments to help facilitate such collaboration.

#### 2.5.1 Approach 1: Dynamical Models

Much of evolutionary theory is based on examining the equilibria of dynamical systems (e.g., mutation‐drift balance) and understanding when perturbations–small changes in the state of the population (e.g., allele frequency, population size)–will result in shifts from one equilibrium to another. This approach is based on the implicit assumption that shifts in ecological conditions and evolutionary parameters are rare, allowing populations to reach an evolutionary equilibrium. However, as exemplified by Prediction 1, urbanization (Figure 1A) often involves disturbance events that occur over short timescales (e.g., colonization, fragmentation), which may outpace the rate at which populations evolve to equilibrium. These changes in the evolutionary parameters (Figure 1B) can move or change the stability of evolutionary equilibria. Dynamical models (e.g., recursion equations or differential equations) following changes in evolutionary quantities, such as heterozygosity, can provide insights into the nature, direction, and speed of transient evolutionary dynamics after such parameter changes.

#### 2.5.2 Approach 2: Simulation‐Based Methods

Theoretical models in evolution often explore the interaction, and ultimately the balance between, contrasting evolutionary processes (e.g., the generation of genetic diversity via mutation and its loss through genetic drift). When multiple evolutionary processes (e.g., mutation, drift, and gene flow) or complex spatial configurations are considered (e.g., Prediction 2), our ability to obtain analytical results (as in Equation 1) can be limited. Simulation‐based approaches serve an important role in this space, allowing us to extend our theoretical tests of verbal hypotheses to include these additional evolutionary processes and/or include system‐specific natural history. Simulations also serve an important role in integrating theoretical and empirical approaches as they can involve the specification of realistic, and ideally empirically derived, parameter values. The application of simulation‐based methods is exemplified in two recent studies by Santangelo et al. (2018) and Savary et al. (2024) that we summarize in Box 1.

#### 2.5.3 Approach 3: Model Contrasts

Urban evolution can be studied empirically by comparing populations in urban and rural environments or theoretically by contrasting models that differ in their underlying ecological or demographic structure. Prediction 3 exemplifies this by comparing inbreeding depression in models with and without population subdivision. This model contrast approach complements the analysis of individual dynamical systems (Approach 1), where the latter considers the effect of changes in model parameters, the former considers the effect of changes in model structure (e.g., the number of state variables or different life histories). Key challenges in formulating model contrasts are defining equivalent model parameters and minimizing the number of differences between the models. For example, the model contrast in Prediction 3 (Whitlock 2002) compares inbreeding depression while holding the total (meta)population size constant and, while certainly not true in many natural systems, assumes that selection is identical in all patches of the sub‐divided metapopulation. Although these assumptions may not be realistic for many natural (meta)populations, they provide a fair model comparison and a baseline to which more complex models can be compared.

#### 2.5.4 Approach 4. Fitness Landscapes

Urban environments exert novel selective pressures on populations—these selective pressures can have many causes and vary in strength and direction (Charmantier et al. 2024). Fitness landscape models, such as Fisher's Geometric Model, can be useful for conceptualizing the complex and multi‐dimensional mapping between genotypes, phenotypes, and fitness in urban populations (Fragata et al. 2019). Theoretical analysis of fitness landscapes has been extraordinarily useful in addressing fundamental questions about adaptation and speciation such as the role of biological constraint, the relative importance of selective and neutral processes, and the predictability of evolution (also see Approach 5). While traditionally studied theoretically, fitness landscape models are increasingly linked to empirical studies either in a top‐down manner—as a way of interpreting empirical results on the predictability of evolution—or bottom‐up—parameterizing fitness landscapes using empirically derived mapping between genotypes or phenotypes and fitness (de Visser and Krug 2014). Fisher's Geometric Model may be particularly useful in urban environments. This model naturally captures adaptation via new mutations under sudden environmental change, as in Prediction 4, and the abstractness of phenotypes and mutations make it particularly easy to adapt to empirical settings (Fragata et al. 2019).

#### 2.5.5 Approach 5. Probabilistic Models

An exceptional feature of cities is their replicated nature, allowing us to assess the repeatability of the evolutionary process in putatively independent populations. As a result of genetic drift, the evolutionary outcome is not guaranteed to be same even in perfectly identical environments. Hence, taking advantage of the replicated nature of cities for formulating and testing evolutionary predictions requires modeling genetic drift in combination with other evolutionary processes in probabilistic models, models that not only consider the expected evolutionary outcome but the full distribution of possible outcomes. These probability distributions are often derived with analytical results from models of genetic drift (e.g., the Wright‐Fisher model and the related diffusion approximation (Crow and Kimura 1970)), which may directly incorporate the effects of experimental sampling (e.g., coalescent‐based results). This is exemplified by Prediction 5 where we considered the probability that the same adaptive allele fixes independently across multiple replicate cities, a result derived from the diffusion approximation. Probabilistic models are often mathematically complex and require simplifying assumptions for tractability. The model used to formulate Prediction 5, for example, makes a number of important simplifying assumptions such as assuming that selection and population sizes are identical across all the cities considered. This is unlikely to be the case in many systems and a comprehensive understanding of the replicability of evolution across urban environments, or lack thereof, will require formulating probabilistic or simulation models that incorporate the observed variation in evolutionary parameters from across cities and explicitly account for non‐independence (e.g., gene flow) between distinct urban areas.

## 3 A Call for Theoreticians

The recent surge of research in urban evolutionary ecology has been dominated by empirical studies (Johnson and Munshi‐South 2017; Rivkin et al. 2019; Szulkin et al. 2020; Miles et al. 2020; Diamond and Martin 2021), with limited contributions from mathematical models. While researchers have put forth conceptual models (Alberti 2015), and formulated predictions based loosely on existing mathematical models (Johnson et al. 2015; Johnson and Munshi‐South 2017; Miles et al. 2019), we are aware of only two theoretical studies offering explicit predictions of how urbanization should influence evolutionary processes (Santangelo et al. 2018; Savary et al. 2024), which we summarize in Box 1. This highlights a significant need for more theoretical work designed to capture the unique biology of urban populations.

BOX 1 **Examples of Simulation Studies Tailored to Understand Urban Evolution.**

Descriptions of two theoretical studies that used simulation approaches to tailor population genetic models to the study of evolution in urban habitats.

**Summary of Santangelo et al. (** **2018**)

**Research Problem:** Previous empirical research documented repeated reductions in the frequency of plants producing hydrogen cyanide (HCN) in urban populations of white clover ( *Trifolium repens* ). However, the epistasis underlying HCN might make populations particularly susceptible to a loss of HCN via purely neutral processes, which may be especially likely in urban populations subject to strong genetic drift.

**Approach:** To assess the role of genetic drift in leading to clines in genetic and phenotypic evolution, Santangelo et al. (2018) simulated the evolution of HCN clines under varying gradients in the strength of genetic drift, gene flow, and natural selection.

**Results and Conclusions:** Genetic drift was able to generate repeated clines in HCN on its own, but these clines were often weak. By contrast, natural selection was necessary to generate clines as strong as those observed across cities, suggesting empirically observed genetic and phenotypic clines are adaptive.

**Summary of Savary et al. (** **2014**)

**Research Problem:** Neutral genetic structure of populations results from the joint effects of genetic drift and gene flow. Yet, disentangling their respective contributions on the evolution of populations is difficult in urban settings where both population size and dispersal typically vary together.

**Approach:** To assess the potential influence of varying levels of gene flow, Savary et al. (2024) simulated population genetic structure within 325 European urban areas, keeping population sizes constant across varying dispersal scenarios.

**Results and Conclusions:** Differences in urban matrix permeability lead to very different spatial genetic patterns. Simulations additionally showed the potential role of source‐sink dynamics for the maintenance and loss of genetic diversity of urban populations and provide neutral expectations for future empirical observations.

Our five predictions, rooted in established theory, represent only a subset of the questions, theoretical models, and approaches that can illuminate ecological and evolutionary processes in cities. Even so, they provide guidance for how existing theory can be applied to empirical research, while simultaneously revealing the opportunity for theory to be tailored to the unique features of cities and how urbanization may alter the evolution of organisms in unique ways. Much of evolutionary theory focuses on longer‐term predictions, equilibrium states, and simplified spatial contexts. In contrast, cities are typically young, rapidly changing, and spatially complex (Seto et al. 2012; Reba et al. 2016; Huang et al. 2019; Mahtta et al. 2019; Liu et al. 2020), so populations are unlikely to be near equilibrium (see Prediction 1). Addressing these gaps with models that are designed to explore shorter‐term transient dynamics would help illuminate the ecological and evolutionary outcomes observed in urban populations, communities, and ecosystems.

Mathematical theory describing eco‐evolutionary dynamics and feedbacks may be particularly well suited to modeling urban systems (Govaert et al. 2019), given that these approaches focus on ecological and evolutionary systems that can rapidly change, as is often the case for urban systems. Spatially explicit simulation models of population genetic and quantitative genetic dynamics may also be well suited to tailoring models to incorporate urban characteristics and the organisms that live in cities. For example, several of the authors of this paper are currently collaborating to implement such approaches in SLiM (Haller et al. 2025) to understand how the age of cities, as well as the size and connectedness of urban parks, interact with species' life‐history characteristics to influence evolutionary and ecological dynamics. We urge theoreticians interested in such topics to advance knowledge in this area by either tailoring existing models to the “urban parameter space” or developing new models relevant to cities. In collaboration with empiricists, such theoretical efforts would allow for the generation of specific hypotheses and predictions that could be empirically tested in cities worldwide. These models could rapidly advance both fundamental evolutionary ecology and its application to the unique socio‐eco‐evolutionary contexts of cities.

## 4 Conclusion

We have highlighted the exciting and critical opportunities available that arise from integrating empirical and theoretical approaches to the study of urban evolutionary biology. Urban evolutionary ecology is a young discipline. To date, research predictions have relied on indirect applications of existing theory adopted from evolutionary biology. For empiricists, our five predictions demonstrate how more precise applications of established theoretical models to urban evolutionary systems can refine predictions, reveal knowledge gaps, and provide insights into fundamental and applied questions. Urban systems offer fertile ground for testing qualitative predictions generated by existing theoretical models at a continuum of spatial scales, and for the application of these predictions to real‐world problems of conservation, restoration, and sustainability. In addition to the empirical tests outlined throughout, the development and tests of quantitative predictions will require additional meta‐analyses assessing the parameter space of urban evolution.

For the theoretician, urban evolutionary ecology offers avenues for the application and tailoring of theoretical models to generate testable predictions in new contexts that often contrast with natural environments. The compressed temporal and spatial scale of urbanization, plus the potential change of the strength and relative importance of evolutionary forces, leads to novel challenges for modelers, since most ecological and evolutionary dynamics in urban areas are unlikely to be anywhere close to a (quasi)equilibrium. Moreover, the constantly changing and complex nature of urban environments means that the ecological and evolutionary forces affecting populations are likely to continuously shift. These challenges can be addressed through collaborations between theoreticians and empirical evolutionary biologists.

## Funding

This work was supported by the School of Cities; Centre for Urban Environments; NSERC Canada Research Chair Program, CRC‐2022‐00111, CRC‐2021‐00276, CRC‐2023‐00139; NSERC Discovery Grant, RGPIN‐2022‐04913, RGPIN‐2025‐06426, RGPIN‐2020‐07188; EEB Postdoctoral Fellowship; Agence Nationale de la Recherche, ANR‐24‐CPJ1‐0035‐01.

## Conflicts of Interest

The authors declare no conflicts of interest.

## Data Availability

The data that support the findings of this study are openly available in GitHub at https://github.com/psavary3/urban_habitat_genetics.
